# Alpha-Ketoglutarate and 5-HMF: A Potential Anti-Tumoral Combination against Leukemia Cells

**DOI:** 10.3390/antiox10111804

**Published:** 2021-11-12

**Authors:** Joachim Greilberger, Ralf Herwig, Michaela Greilberger, Philipp Stiegler, Reinhold Wintersteiger

**Affiliations:** 1 Division of Physiological Chemistry, Otto Loewi Research, Medical University of Graz, 8010 Graz, Austria; 2 Schwarzl Medical Center, Institute of Scientific Laboratory, 8053 Graz, Austria; institut@laborwissenschaft.at; 3 German Medical Center, Department of Urology Surgery, Dubai 665664, United Arab Emirates; Ralf.herwig@gmcdhcc.com; 4 Men’s Health Clinic, 80337 Munich, Germany; 5 Division of Transplantation Surgery, Medical University of Graz, 8010 Graz, Austria; philipp.stiegler@medunigraz.at; 6 Department of Pharmaceutical Chemistry, Institute of Pharmaceutical Sciences, University of Graz, 8010 Graz, Austria; reinhold.wintersteiger@uni-graz.at

**Keywords:** alpha-ketoglutarate (aKG), 5-hydroxy-methyl-furfural (5-HMF), reactive oxygen and nitrogen species (RONS), leukemia, human fibroblasts (HF-SAR), proliferation, caspase activity, carbonylated proteins (CP)

## Abstract

We have recently shown that a combined solution containing alpha-ketoglutarate (aKG) and 5-hydroxymethyl-furfural (5-HMF) might have anti-tumoral potential due to its antioxidative activities. The question arises if these substances have caspase-3- and apoptosis-activating effects on the cell proliferation in Jurkat and HF-SAR cells. Antioxidative capacity of several combined aKG + 5-HMF solution was estimated by cigarette smoke radical oxidized proteins of fetal calf serum (FCS) using the estimation of carbonylated proteins. The usage of 500 µg/mL aKG + 166.7 µg/mL 5-HMF showed the best antioxidative capacity to inhibit protein modification of more than 50% compared to control measurement. A Jurkat cell line and human fibroblasts (HF-SAR) were cultivated in the absence or presence of combined AKG + 5-HMF solutions between 0 µg/mL aKG + 0 µg/mL 5-HMF and different concentrations of 500 µg/mL aKG + 166.7 µg/mL 5-HMF. Aliquots of Jurkat cells were tested for cell proliferation, mitochondrial activity, caspase activity, apoptotic cells and of the carbonylated protein content as marker of oxidized proteins in cell lysates after 24, 48, and 72 h of incubation. The combined solutions of aKG + 5-HMF were shown to cause a reduction in Jurkat cell growth that was dependent on the dose and incubation time, with the greatest reductions using 500 µg/mL aKG + 166.7 µg/mL 5-HMF after 24 h of incubation compared to 24 h with the control (22,832 cells vs. 32,537 cells), as well as after 48 h (21,243 vs. 52,123 cells) and after 72 h (23,224 cells). Cell growth was totally inhibited by the 500 µg/mL AKG + 166.7 µg/mL solution between 0 and 72 h of incubation compared to 0 h of incubation for the control. The mitochondrial activity measurements supported the data on cell growth in Jurkat cells: The highest concentration of 500 µg/mL aKG + 166.7 µg/mL 5-HMF was able to reduce the mitochondrial activity over 24 h (58.9%), 48 h (28.7%), and 72 h (9.9%) of incubation with Jurkat cells compared not only to the control incubation, but also to the concentrations of 500 µg/mL aKG + 166.7 µg/mL 5-HMF or 375 µg/mL aKG 125 µg/mL 5-HMF, which were able to significantly reduce the mitochondrial activity after 48 h (28.7% or 35.1%) and 72 h (9.9% or 18.2%) compared to 24 h with the control (100%). A slight increase in cell proliferation was found in HF-SAR using the highest concentration (500 µg/mL aKG + 166.7 µg/mL 5-HMF) between 0 h and 72 h incubation of 140%, while no significant differences were found in the mitochondrial activity of HF-SAR in the absence or presence of several combined aKG + 5-HMF solutions. The solutions with 500 µg/mL aKG + 166.7 µg/mL 5-HMF or 250 µg/mL aKG + 83.3 µg/mL 5-HMF showed a significantly higher caspase activity (51.6% or 13.5%) compared to the control (2.9%) in addition to a higher apoptosis rate (63.2% or 31.4% vs. control: 14.9%). Cell lysate carbonylated proteins were significantly higher in Jurkat cells compared to HF-SAR cells (11.10 vs. 2.2 nmol/mg). About 72 h incubation of Jurkat cells with 500 µg/mL aKG + 166.7 µg/mL 5-HMF or 250 µg/mL aKG + 83.3 µg/mL 5-HMF reduced significantly the carbonylated protein content down to 5.55 or 7.44 nmol/mg whereas only the 500 µg/mL aKG + 166.7 µg/mL 5-HMF solution showed a significant reduction of carbonylated proteins of HF-SAR (1.73 nmol/mg).

## 1. Introduction

Clinical and pre-clinical studies of an anti-tumoral solution containing aKG, 5-HMF, N-acetyl-selenomethionine, and N-acetylmethionine for treating tumors [1,2] showed, on one hand, good therapeutic efficacy during infusion therapy in prostate cancer patients by increasing the PSA doubling time; on the other hand, a reduction of tumoral mass was shown in lung cancer patients [1].

The reduction of oxidatively modified proteins generated by cigarette smoke [3] demonstrated the impressive effects of aKG + 5-HMF as a better potential antioxidative solution compared to vitamin C or its single compounds. aKG itself is not only involved in the energy generation process in humans, but also in several metabolic processes for enzymatic regulation, such as those of hypoxia-inducing factor alpha [4] or 2-oxo-glutarate-dependent dioxygenases in cancer [5] and to suppress tumors in bladder cancer patients [5,6,7].

The compound 5-HMF occurs in honey and apple juice and in even higher rates in dried fruits, caramel products, and coffees [8]. Because there was speculation that 5-HMF is cancerogenic, the National Institute of Environmental Health Sciences demonstrated that no evidence of any carcinogenic activity was found when applying concentrations of 750 mg/kg over 2 years in rats and also provided some evidence in mice. Anti-proliferative and antioxidative activities were found in 5-HMF, suggesting its potential chemoprevention in cancer [9] as well as in melanoma cells [10]. aKG + 5-HMF was demonstrated to increase oxygen saturation during exercise in subjects with normobaric hypoxia [11] because of the antioxidative and anti-sickling effects of 5-HMF and its increased affinity for oxygen [12].

Given these reports, the present study was intended to check whether the addition of a combined solution of aKG + 5-HMF to leukemic cells would exert anti-tumoral, anti-proliferative, and apoptotic effects compared to HF-SAR.

## 2. Materials and Methods

### 2.1. Materials

Media: RPMI 1640, FBS, DMEM, antibiotic–antimycotic solution (Thermofisher, Vienna, Austria); reagents: Cytofix–Cytoperm permeabilization Kit (Thermofisher, Vienna, Austria), FITC Active Caspase-3 Apoptosis Kit (BD Biosciences Kit; Allschwil, Germany), WST-1 Cell Proliferation Reagent (Abcam; Cambridge, UK); chemicals: alpha-ketoglutarate (Sigma), 5_hydroxymethyl-furfurale (5-HMF, Evonik Operation, Darmstadt, Germany), guanidine-HCl, butyl-hydroxy-toluene (BHT; Sigma Aldrich), di-nitro-phenyl-hydrazine (DNPH) (Altmann Analytics, Munich, Germany), and flasks (Falco^®^ Cell Culture; Corning Incorporated, 14831 New York, NY, USA) were used.

### 2.2. Oxidatively Modified Protein Measurements of FCS by Cigarette Smoke Radicals

In vitro protein damage of FCS proteins (=control measurement) by cigarette smoke was assessed according to this setup (4): 50 mg/mL serum protein was dissolved in phosphate buffered saline. Total of 4 mL of protein solution was transferred to a suction bottle, which was connected to a water pump. Before smoking, two 100 µL aliquots of protein-solution were diluted with 1150 µL 10 mM PBS, pH 7.4, containing 40 µM BHT to obtain a 4 mg/mL BSA solution. About 1 mg of protein was precipitated by pipetting 250 µL of the 4 mg/mL protein solution with 250 µL 20% trichloric acid (TCA) solution. After smoking within 2 min further aliquots were diluted and precipitated with TCA. The glass was closed and incubated at 37 °C. After 15, 30, and 60 min aliquots were taken out, diluted, and precipitated. All precipitated samples (1 mg) were centrifuged at 5000× *g* for 3 min and the supernatant was removed. Pellets (1 mg) were dissolved in 10 mM DNPH containing 6 M guanidine pH 2.5 and incubated for 45 min at room temperature. Protein damage was measured spectro-photometically with the protein carbonyl assay according to Levine et al. [13]. Protein carbonyl data are expressed in nmol/mg.

### 2.3. Cell Culture

The Jurkat-J6 cell line was procured from the National Center for Cell Sciences (Pune, India), and it had confluency > 80%. It is an immortalized line of human T-lymphocyte cells derived from acute T-cell leukemia. It is used to study acute T-cell leukemia. Jurkat-J6 cell line has the ability to produce IL-2 and that is why it is useful for different anti-cancer study [14]. It was an immortalized line of human T-lymphocyte cells derived from acute T-cell leukemia. The Jurkat-J6 cell line was centrifuged, re-suspended in complete RPMI-1640 + glutamine media (containing 10% FBS and 1% antibiotic–antimycotic solution) in flasks, and incubated at 37 °C with a continuous 5% CO_2_ supply. The cell density was adjusted to about 1–2 × 10^6^ cells/mL for all of the further experiments. The HF-SAR cell line was established from the normal human skin of a 2-year-old male [15]. The HF-SAR cell line was centrifuged, re-suspended in complete DMEM–high-glucose media (containing 10% FBS and 1% antibiotic–antimycotic solution) in flasks, and incubated at 37 °C with a continuous 5% CO_2_ supply. The cell density was adjusted to about 1 × 10^6^ cells/mL for the estimation of mitochondrial activity.

### 2.4. Cell Proliferation Experiments

Cell proliferation experiments were carried out by incubating different aKG/5-HMF combinations (0 µg aKG + 0 µg/mL 5-HMF, 125 µg aKG + 41.7 5-HMF µg/mL, 200 µg aKG + 66.7 µg 5-HMF µg/mL, 250 µg AKG + 83.3 µg/mL 5-HMF, 375 µg aKG + 125.4 µg/mL 5-HMF, and 500 µg aKG + 166.7 µg/mL 5-HMF) in an appropriate medium for 24, 48, and 72 h with the cells. The cell proliferation was estimated with the CASY^®^ Cell Counter (Hoffmann-La Roche Ltd., Basel, Switzerland). Aliquots were used for further experiments.

### 2.5. Cytotoxicity Assay

A cytotoxicity assay (WST assay) was carried out to determine the viability of the Jurkat and HF-SAR cell lines. The WST-1 Cell Proliferation Reagent provided a simple and accurate method for measuring cell proliferation. 

The assay was carried out as described in the manufacturer’s instructions. In brief, cells (200,000/mL) were seeded onto transparent 24-well plates and incubated for 0, 24, 48, and 72 h in the absence or presence of an aKG + 5-HMF combination (0 µg/mL aKG + 0 µg/mL 5-HMF, 125 µg/mL aKG + 41.7 µg/mL 5-HMF, 200 µg/mL aKG + 66.7 µg/mL 5-HMF, 250 µg/mL aKG + 83.3 µg/mL 5-HMF, 375 µg/mL aKG + 125 µg/mL 5-HMF, and 500 µg/mL aKG + 166.7 µg/mL 5-HMF). The cells were then washed twice with DPBS 1X and incubated in a fresh medium with 10% WST-1 reagent for 2 h. The absorbance was measured at 450 nm (690 nm was used as a reference wavelength and subtracted) in a spectrophotometric reader (Spectra Max Pro 384; Molecular Devices; 95134 San Jose, CA, USA). 

### 2.6. Caspase-3 Activity Measurements

After incubation for 72 h in the absence of any substances (negative control), in the presence of 4 or 0.4 µM CPT (positive control), or in the presence of either 250 µg/mL aKG + 83.3 µg/mL 5-HMF or 500 µg/mL aKG + 166.7 µg/mL 5-HMF, a population of 1.0 × 10^6^ cells/mL was centrifuged (350× *g*) and re-suspended in PBS; the supernatant was removed, and the cells were washed again with cold PBS and then centrifuged (350× *g*). After removing the supernatant, the pellet was re-suspended in 500 µL of Cytofix–Cytoperm solution and incubated on ice for 20 min. 

After the washing and centrifugation steps and the use of 500 µL of Perm/Wash buffer three times, the samples containing 100 µL of Perm/Wash buffer were incubated with 20 µL of antibody–FITC for 30 min in the dark. After incubation, the samples were washed with Perm/Wash buffer and centrifuged (350× *g*); the supernatant was removed, and the samples were re-suspended in the Perm/Wash buffer three times. The FITC–Caspase-3 activity was measured on an FACS Calibur^®^. The supernatant was collected, and 50 μL of reaction buffer and 5 μL of DEVD-AFC (final concentration of 50 μM) were added and further incubated for 2 h at 37 °C. The resultant fluorescence was measured by using excitation and emission wavelengths of 495 and 519 nm, respectively.

### 2.7. Detection of the Mitochondrial Membrane Potential with Flow Cytometry

After incubation for 72 h in the absence of any substances (negative control), in the presence of 4 or 0.4 µM CPT (positive control), or in the presence of either 250 µg/mL aKG + 83.3 µg/mL 5-HMF or 500 µg/mL aKG + 166.7 µg/mL 5-HMF, a population of 2.0 × 10^6^ cells/mL was centrifuged (400 g), re-suspended in 0.5 mL of JC-1 solution (5,5′,6,6′-tetracholor-1,1′,3,3′-tertaehtylbenzimidazolcarbocyanin-iodide; 125 µL stock solution + 12.375 mL assay buffer), and incubated for 15 min and with 5% CO_2_ at 37 °C. After two washing steps with the assay buffer (BD Biosciences Kit; 4123 Allschwil, Germany), the cells were re-suspended in 1 mL of assay buffer and analyzed with FACS. The aggregate dye was able to excite the monomer at 535 nm and the aggregate at 475 nm. 

### 2.8. Cell Lysate Protein Damage Measurements 

Protein concentration of cell lysates were estimated according to the BSA Test Kit. About 25 µL of diluted albumin standards and cell lysates were transferred into 96 microtitration plates for triple estimations. After addition of the BCA reagent solution (50:1) the microtitration plate was covered with a top cover and incubated for 30 min on a plate shaker at 37 °C with a continuous 5% CO_2_ supply. After cooling to room temperature, the content of proteins were estimated at 562 nm (Spectra Max Photometer Pro 384). Samples were diluted to 1 mg/mL protein for the estimation of carbonylated proteins described above.

### 2.9. Statistical Analysis

Group comparisons were made by using *t*-tests where appropriate and indicated. Linear regression and exponential regression curves were calculated based on Pearson regression (SPSS 25, SPSS Inc., Chicago, IL, USA). All values are given as the mean values and standard deviations. Statistical significance was considered to be at *p* < 0.01, with high significance at *p* < 0.001.

## 3. Results

### 3.1. Estimation of Different AKG/5-HMF Ratios during Exposure of Cigarette Smoke on FCS Proteins

**Figure 1:**
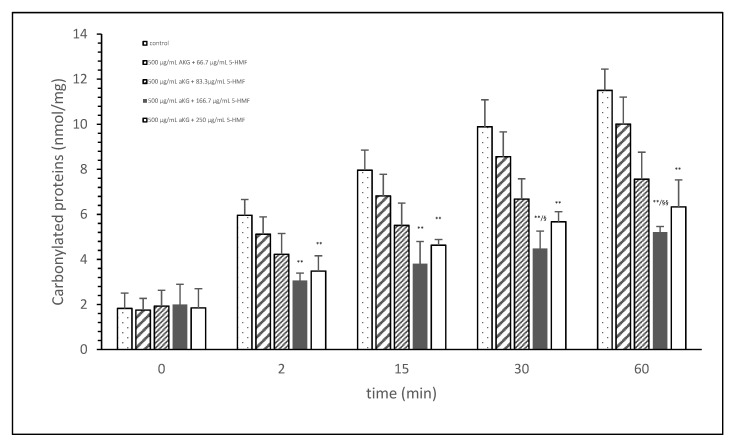
Cigarette smoke oxidatively modified proteins of FCS after cigarette smoke exposure in presence or absence of different AKG and 5-HMF combined solutions expressed with the content of carbonylated proteins (*n* = 3). ** *p* < 0.01: significance between the control (0 µg/mL aKG + 0 µg/mL 5-HMF) and different combinations of aKG + 5-HMF after 2, 15, 30, and 60 min exposure. ^§^ *p* < 0.05: significance between the 500 µg/mL aKG + 83.3 µg/mL 5-HMF and 500 µg/mL aKG + 125 µg/mL 5-HMF. ^§§^ *p* < 0.01: significance between the 500 µg/mL aKG + 83.3 µg/mL 5-HMF and 500 µg/mL aKG + 125 µg/mL 5-HMF.

Figure 1 shows the oxidative modification of FCS protein after 2, 15, 30, and 60 min of exposure of cigarette smoke expressed with carbonylated proteins (nmol/mg protein) using different AKG+5-HMF combination solutions. The best significant reduction of carbonyl proteins was found using the 500 µg/mL + 125 µg/mL 5-HMF solution compared to control. After 2 min the carbonylated protein was significantly lower (3.06 ± 0.33 vs. 5.96 ± 0.70 nmol/mg; *p* < 0.01), also after 15 min (3.80 ± 0.99 vs. 7.96 ± 0.33 nmol/mg; *p* < 0.01), 30 min (4.49 ± 0.77 vs. 9.89 ± 1.20 nmol/mg; *p* < 0.01), and 60 min (11.51 ± 0.94 vs. 5.21 nmol/mg; *p* < 0.01). At time points 30 and 60 min, this solution (500 µg/mL + 125 µg/mL 5-HMF) also showed a significantly lower carbonylated protein content compared to 500 µg/mL aKG + 62.5 µg/mL 5-HMF (6.68 ± 0.90 nmol/mg, *p* < 0.05 and 7.56 ± 1.20 nmol/mg, *p* < 0.01). The highest used combination 500 µg/mL + 250 µg/mL 5-HMF showed no significant difference compared to 500 µg/mL + 125 µg/mL 5-HMF, but the carbonyl proteins were higher with the highest combination solution. For the following experiments we have used the 500 µg/mL aKG + 166.7 µg/mL 5-HMF solution and its dilutions.

### 3.2. Cell Proliferation Experiments

**Figure 2:**
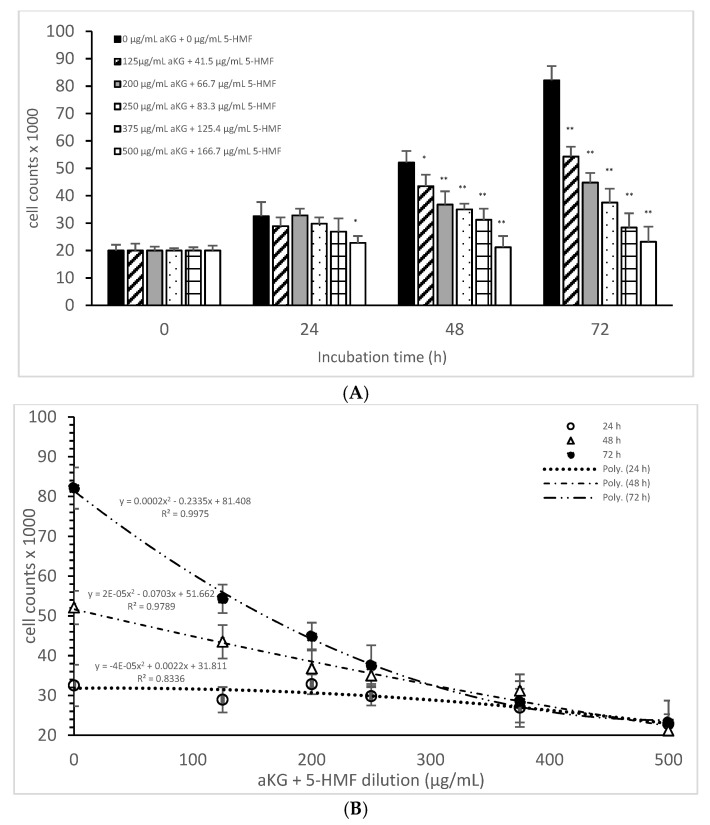
Cell growth of the Jurkat cell line in the absence or presence of different concentrations of the combined aKG + 5-HMF (**A**,**B**) correlation between cell growth and the combined solutions of aKG + 5-HMF after 24, 48, and 72 h of cultivation (*n* = 5). * *p* < 0.05: significance between the control (0 µg/mL aKG + 0 µg/mL 5-HMF) and different combinations of aKG + 5-HMF at the time points after 1, 2, and 3 days of cell culture. ** *p* < 0.001: significance between the control (0 µg/mL aKG + 0 µg/mL 5-HMF) and different combinations of aKG + 5-HMF at the time points after 1, 2, and 3 days of cell culture.

Figure 2A describes the cell growth with different combinations of aKG + 5-HMF in Jurkat cells over 3 days. After 24 h, only the highest concentration (500 µg/mL aKG and 166.7 µg/mL 5-HMF) showed a significant reduction in cell growth compared to the control at 24 h (22,832 ± 2512 cells vs. 32,537 ± 5231 cells; *p* < 0.05). No significant changes were estimated between the control at 0 h and the Jurkat cells incubated in the presence of 500 µg/mL aKG + 166.7 µg/mL 5-HMF for 24 h. The cell growth at 48 h was significantly reduced compared to the control (52,123 ± 4232 cells, *n* = 5) by several different concentrations of the combination of aKG + 5-HMF: 125 µg/mL aKG + 41.7 µg/mL 5-HMF (43,511 ± 4209 cells; *p* < 0.05; *n* = 5), 200 µg/mL aKG + 66.7 µg/mL 5-HMF (36,823 ± 4845 cells; *p* < 0.001; *n* = 5), 250 µg/mL aKG + 83.3 µg 5-HMF (35,098 ± 2150 cells; *p* < 0.001; *n* = 5), 375 µg/mL aKG + 125 µg/mL 5-HMF (31,245 ± 4111 cells; *p* < 0.001; *n* = 5), and 500 µg/mL aKG + 166.7 µg/mL 5-HMF (21,243 ± 55,467 cells; *p* < 0.001; *n* = 5). After 72 h of incubation, the greatest combination, 500 µg/mL aKG + 166.7 µg/mL 5-HMF (23,224 ± 5445 cells; *p* < 0.001; *n* = 5), showed a significant reduction compared to the control after 72 h (82,131 ± 5197 cells; *p* < 0.001; *n* = 5), but did not show a reduction compared to the control cells after 0 or 24 h. A lesser reduction in the cell growth compared to the control after 72 h were obtained with 375 µg/mL aKG + 125 µg/mL 5-HMF (28,433 ± 5247 cells; *p* < 0.001; *n* = 5), 250 µg/mL aKG + 83.3 µg/mL 5-HMF (37,512 ± 5129 cells; *p* < 0.001; *n* = 5), 200 µg/mL aKG + 66.7 µg/mL 5-HMF (44,768 ± 3487 cells; *p* < 0.001; *n* = 5), and 125 µg/mL aKG + 41.7 µg/mL 5-HMF (54,227 ± 3655 cells; *p* <0.05; *n* = 5).

After correlating several concentrations of the combined AKG + 5-HMF solutions with cell growth (Figure 2B), all three curves showed a high polynomial correlation. The best correlation was calculated with the cell growth after 72 h of incubation (r = nearly 1; y = 0.0002x^2^ − 0.2335x + 81408) and the IC50% calculated for the 100 µg/mL AKG + 41.7 µg/mL 5-HMF solution, followed by 48 h of incubation (r = 0.99; y = 2 × 10^−5^x^2^ − 0.0703x + 51662) and the IC50% of the 200 µg/mL aKG + 66.7 µg/mL 5-HMF solution, and finally, the 24-h incubation (r = 0.91; y = y = −4 × 10^−5^x^2^ + 0.0022x + 31811) and the IC50% of around 375 µg/mL aKG + 125 µg/mL 5-HMF. These results showed also that the higher the incubation time with AKG + 5-HMF the lower concentrations are needed to reach the IC 50%. The decline of cell growth after 72 h incubation was higher compared to the 48 h and 24 h incubation.

Table 1 shows the cell proliferations (%) of Jurkat cells and HF-SAR cells after 0, 24, 48, and 72 h incubation in presence or absence of the combined solutions of aKG + 5-HMF. No significant difference was estimated between all used aKG + 5-HMF solutions during all incubations except with the highest concentration 500 µg/mL aKG + 166.7 µg/mL 5-HMF after 72 h incubation (*p* < 0.05) compared to 0, 24, and 48 h incubation.

### 3.3. Cytotoxic Assay

**Figure 3:**
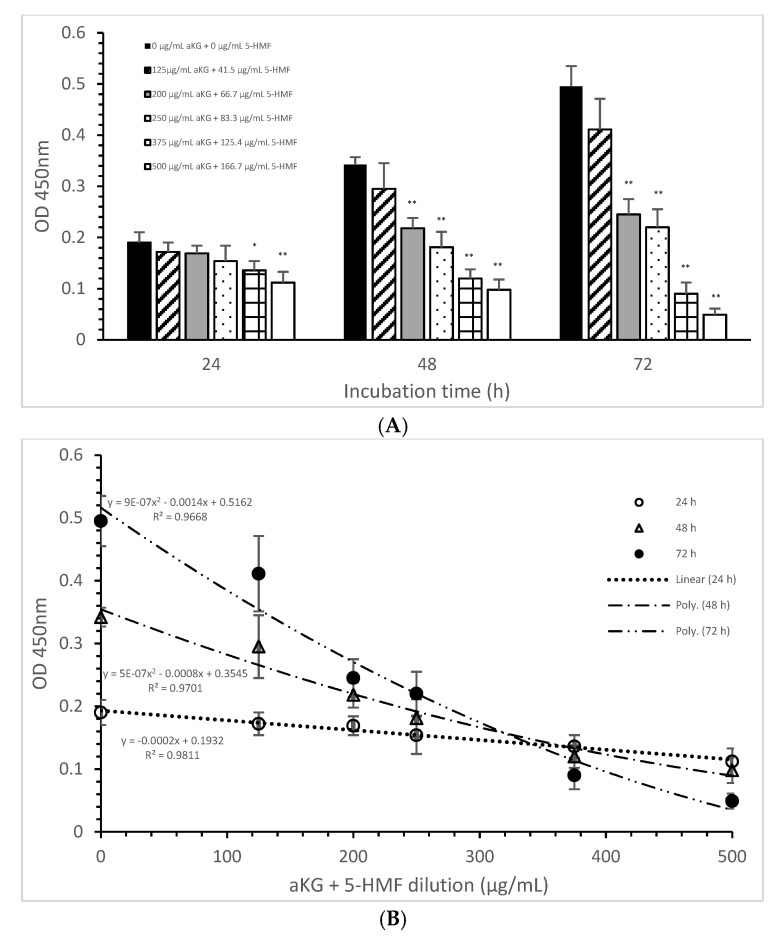
Mitochondrial activity of the Jurkat cell line in the absence or presence of different aKG + 5-HMF concentrations (**A**); (**B**) correlation between mitochondrial activity and the combined aKG + 5-HMF concentration after 24, 48, and 72 h of cultivation (*n* = 5). * *p* < 0.01: significance between the control (0 µg/mL aKG + 0 µg/mL 5-HMF) and different combinations of aKG + 5-HMF at time points after 24, 48, and 72 h of cell culture. ** *p* < 0.001: significance between the control (0 µg/mL aKG + 0 µg/mL 5-HMF) and different combinations of aKG + 5-HMF at time points after 1, 2, and 3 days of cell culture.

The mitochondrial activity of the Jurkat cells as it was expressed in the absorbance at 450 nm in the absence or presence of one of the dilutions of the aKG + 5-HMF solution is presented in Figure 3A. After 24 h of incubation, the highest concentration (500 µg/mL aKG and 166.7 µg/mL 5-HMF (0.112 ± 0.021)) showed a 41% reduction of the mitochondrial activity, which was significant compared to that of the control after 24 h (0.19 ± 0.02; *n* = 5; *p* < 0.001). Using the 375 µg/mL aKG + 125 µg/mL 5-HMF solution (0.136 ± 0.018; *n* = 5; *p* < 0.05) resulted in a 28% reduction compared to the control after 24 h.

The use of 48 h of incubation resulted in a higher reduction (of 36%) of the mitochondrial activity compared to that of the control after 48 h, with the 500 µg/mL aKG and 166.7 µg/mL 5-HMF solution (0.098 ± 0,02; *n* = 5; *p* < 0.001) showing the greatest reduction, followed by the 375 µg/mL aKG + 125 µg/mL 5-HMF solution (0.120 ± 0.018; *n* = 5; *p* < 0.001), the 250 µg/mL aKG + 83.3 µg 5-HMF solution (0.181 ± 0.02; *n* = 5; *p* < 0.001), and the 200 µg/mL aKG + 66.7 µg/mL 5-HMF solution (0.218 ± 0.05; *n* = 5; *p* < 0.001). The lowest concentration, 125 µg/mL aKG + 41.7 µg/mL 5-HMF (0.295 ± 0.05; *n* = 5), showed no effects. The same trend could be seen after 72 h of incubation. While no effects compared to the control (0.495 ± 0.04) after 72 h were obtained when using 125 µg/mL aKG + 41.7 µg/mL 5-HMF (0.411 ± 0.06; *n* = 5), all the other used concentrations showed significant reductions: 200 µg/mL aKG + 66.7 µg/mL 5-HMF (0.245 ± 0.03; *n* = 5; *p* < 0.001), 250 µg/mL aKG + 83.3 µg 5-HMF (0.222 ± 0.035; *n* = 5; *p* < 0.001), 375 µg/mL aKG + 125 µg/mL 5-HMF (0.090 ± 0.022; *n* = 5; *p* < 0.001), and 500 µg/mL aKG and 166.7 µg/mL 5-HMF (0.098 ± 0.02; *n* = 5; *p* < 0.001). The mitochondrial activity in the Jurkat cells incubated for 72 h in the presence of 500 µg/mL aKG and 166.7 µg/mL 5-HMF was significantly lower than that with 48 and 24 h of incubation when using the same combined concentration (*p* < 0.001). A significant difference was also estimated between incubation for 72 h (0.090 ± 0.022; *n* = 5; *p* < 0.001) and incubation for 24 h (0.136 ± 0.018) with the use of 375 µg/mL aKG + 125 µg/mL 5-HMF.

Figure 3B shows the correlations between the combined solutions of aKG + 5-HMF and the mitochondrial activity at different incubation times with nearly equal regression terms: r = 0.98 for 72 h with a polynomial function (y = 9 × 10^−7^ x^2^ − 0.0014x + 0.5162), r = 0.98 for 48 h with a polynomial function (y = 5 × 10^−7^ x^2^ − 0.0008x + 0.3545), and r = 0.99 for 24 h with a linear function (y = −0.0002x + 0.1932). The IC50% was calculated for all functions, with nearly the same result of 250 µg/mL + 83.3 µg/mL 5-HMF. The decline of the mitochondrial activity was higher during 72 h incubation followed by 48 h incubation compared to 24 h incubation because of its different functions. The usage of 500 µg/mL aKG and 166.7 µg/mL 5-HMF and 375 µg/mL aKG + 125 µg/mL 5-HMF solutions showed a lower mitochondrial activity in favor of 72 h incubation followed by 48 h incubation compared to 24 h incubation.

Table 2 shows the decrease in the mitochondrial activity in the presence of the combined solutions (aKG + 5-HMF). The longer the incubation time and the higher the concentration of aKG + 5-HMF, the lower the mitochondrial activity was. A reduction of nearly half was obtained by using 200 µg/mL aKG + 66.7 µg/mL 5-HMF after 72 h of incubation or by using 250 µg/mL aKG + 83.3 µg/mL 5-HMF after 48 and 72 h of incubation.

Mitochondrial activity at a level of nearly 30% was obtained after 48 h of incubation by using 375 µg/mL + 125.4 µg/mL 5-HMF and 500 µg/mL aKG + 166.7 µg/mL 5-HMF. Mitochondrial activity at levels of 10% and 20% remained after 72 h of incubation with 500 µg/mL aKG + 166.7 µg/mL 5-HMF and after 72 h of incubation with 375 µg/mL aKG + 125.4 µg/mL 5-HMF, respectively.

The incubation of HF-SAR did not result in relevant differences between the different concentrations of aKG + 5-HMF and the incubation times compared to the control (Table 2).

### 3.4. Caspase-3 Activity Measurements

**Figure 4:**
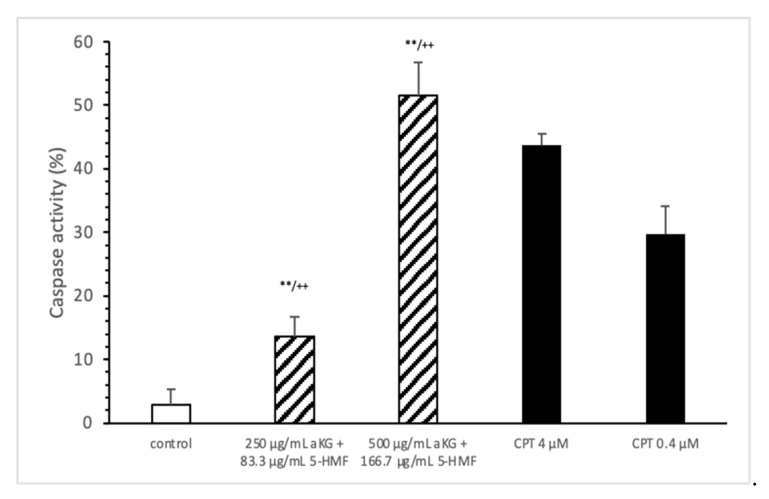
Caspase activity of the Jurkat cell lines after 72 h of cell growth using combined solutions of 250 µg/mL aKG + 83.3 µg/mL 5-HMF solution, 500 µg/mL aKG + 166.7 µg/mL 5-HMF, and 4 or 0.4 µM CPT as positive controls (*n* = 3). ** *p* < 0.01: significance between the control and combined solutions of aKG + 5-HMF. ^++^ *p* < 0.001: significance between the positive control (4 µM CPT) and combined solutions of aKG + 5-HMF.

The loss of mitochondrial activity mostly induces caspase activity (Figure 4). Compared to the control (2.9 ± 2.3%), 250 µg/mL aKG + 83.3 µg/mL 5-HMF significantly increased the caspase activity in Jurkat cells after 72 h of incubation (13.5 ± 3.2; *n* = 3; *p* < 0.001), but 500 µg/mL aKG + 166.7 µg/mL 5-HMF did so even more (51.6 ± 5.2%; *n* = 3; *p* < 0.001). While the activity with 500 µg/mL + 166.7 µg/mL 5-HMF was significantly higher than that of the positive control with 4 µM CPT (43.8 ± 1.8; *n* = 3; *p* < 0.01), that of 250 µg/mL aKG + 83.3 µg/mL 5-HMF was lower (13.5 ± 3.2, *n* = 3; *p* < 0.001).

### 3.5. Detection of the Mitochondrial Membrane Potential through Flow Cytometry

**Figure 5:**
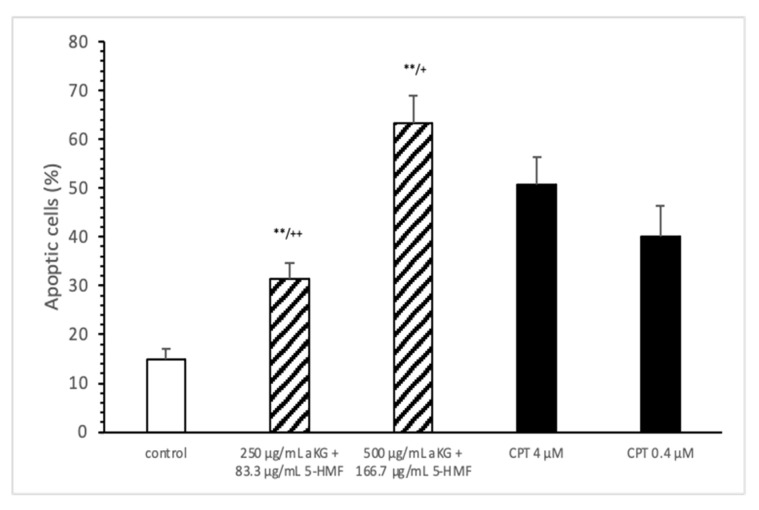
Estimation of apoptotic cells (JC-1) of the Jurkat cell lines after 72 h of cell growth using a combined solutions of 250 µg/mL aKG + 83.3 µg/mL 5-HMF, 500 µg/mL aKG + 166.7 µg/mL 5-HMF, and 4 µM or 0.4 µM CPT as positive controls (*n* = 3). ** *p* < 0.001: significance between the control and combined solutions of aKG + 5-HMF. ^+^ *p* < 0.01: significance between the positive control (4 µM CPT) and combined solutions of aKG + 5-HMF. ^++^ *p* < 0.001: significance between the positive control (4 µM CPT) and combined solutions of aKG + 5-HMF.

The estimation of apoptotic cells (Figure 5) was significantly increased with 250 µg/mL aKG + 83.3 µg/mL 5-HMF after 72 h of incubation (31.4 ± 3.2%) compared to the control (14.9 ± 2.2%; *n*=3; *p* < 0.001), but was significantly decreased compared to 4 µM CPT (50.7 ± 5.6%). The 500 µg/mL aKG + 166.7 µM 5-HMF combination showed a higher significance (63.2 ± 5.6%; *n* = 3; *p* < 0.001) compared to the control and to 4 µM CPT (*n* = 3; *p* < 0.05).

### 3.6. Estimation of Carbonylated Proteins in Jurkat and HF-Sar Cells 

After 72 h incubation with 250 µg/mL aKG + 83.3 µg/mL 5-HMF the carbonylated protein content of isolated membrane proteins of Jurkat cell line was significantly lower compared to 0 h (11.6 ± 0.67 vs. 7.44 ± 0.93 nmol/mg; *p* < 0.01), but not of HF-SAR as presented in Table 3. Using 500 µg/mL aKG + 166.7 µg/mL 5-HMF the carbonylated protein level showed a significant reduction (10.6 ± 0.37 vs. 5.55 ± 1.22; *p* < 0.01) in Jurkat cells and also in HF-SAR (2.5 ± 0.6 vs. 1.73 ± 0.52 nmol/mg; *p* < 0.05). Furthermore, the carbonylated protein content of Jurkat lysates showed a significantly higher content (11.1 ± 0.70 nmol/mg) compared to HF-SAR lysate (2.30 ± 0.66 nmol/mg; *p* < 0.01) before incubation with 250 µg/mL aKG + 83.3 µg/mL 5-HMF or 500 µg/mL aKG + 166.7 µg/mL 5-HMF.

## 4. Discussion

Carbonyl-related substances, such as aldehydes and ketones, are upcoming candidates for the development of anticancer drugs, and they have received extensive research attention. Some of them, such as cinnamaldehydes [16], alpha-ketoglutarate [17], and 5-HMF [18], have oxidative and anti-oxidative potential, which contributes to their potential in chemotherapy. In a previous work we found an antioxidative potential against protein modification of its single compounds aKG and 5-HMF against cigarette smoke radicals, but much better in a combined form [3]. It is suggested that this combination of both substances does react synergistically, but the optimal ratio of both substances to inhibit or reduce oxidative stress was not clear. Here, we present data from several combined aKG + 5-HMF combinations, which showed 500 µg/mL aKG + 166.7 µg/mL 5-HMF for the best prevention solution of oxidizing proteins during exposure of cigarette smoke radicals.

aKG is a new and interesting substance not only for mitochondrial energy metabolism, but also for enzymatic reactions as a substrate or co-substrate. Little is known about it as anti-tumor acting substance, but we showed its anti-tumoral effects on lung cancer patients both in vivo and in vitro, as well as on cancer cells in a novel anti-tumoral solution combining aKG and 5-HMF [1,2]. αKG is the obligate co-substrate of Fe(II)/2-oxoglutarate-dependent dioxygenases (OGDD), a superfamily of enzymes that catalyzes the oxidative decarboxylation of αKG, producing succinate and CO_2_ from O_2_ [19]. Prolyl hydroxylation of hypoxia-inducible factor (HIF)-α, as catalyzed by the Fe(II)/2-oxoglutarate (AKG)-dependent prolyl hydroxylase domain (PHD) enzymes, has a hypoxia-sensing role in animals [20].

Furthermore, the binding of prolyl-hydroxylated HIF-α to PHD2 is ~50-fold hindered by prior αKG binding; thus, when αKG is limiting, HIF-α degradation might be inhibited by PHD binding [20]. Given that αKG is a limiting co-substrate for PHD activity during normoxia and that 2-oxoglutarate (αKG) levels depend on amino acid availability, it is possible that PHD activity depends not only on oxygen but also on amino acid availability. This suggests a global metabolic sensor function for PHDs, which could be signaling not only to HIF-α but also to mTOR [21]. 5-HMF was investigated to have in vitro antioxidative activity by scavenging ABTS and DPPH free radicals in a dose-dependent manner [9,18].

Here, we present the first data on these two main compounds—aKG + 5-HMF—which were combined in several dilutions of the 500 µg/mL aKG + 166.7 µg/mL 5-HMF combination and applied to Jurkat cells, as well as to HF-SAR as a non-cancer cell line. We want to point out that the mitochondrial activity was neither negatively nor positively affected by the combined aKG + 5-HMF solutions in the HF-SAR cells within 24, 48, or 72 h of incubation. The slight cell proliferation obtained after 72 h incubation with the highest aKG + 5-HMF combination might be an effect from aKG because of its positive effect on mitochondrial activity, but also as an antioxidant in preventing peroxide generation during incubation in cell media like RPMI or DMEM alone and in combination with cell culture [22]. Furthermore, vitamin C was suggested to promote peroxide generation in media with or without cell culture. 5-HMF does have a likewise structure and antioxidative activity as vitamin C and therefore also is able to produce in media peroxides. Therefore, the protection of aKG against media-generated peroxides, like hydrogen peroxide and peroxynitrite, is suitable to stabilize 5-HMF [23,24] as we have seen that the carbonylated protein content of cell lysate was decreased in HF-SAR but also in Jurkat cells during incubation with aKG and 5-HMF.

Until now, 5-HMF had been shown to have cancerogenic effects, but this was mostly disproved by the National Institute of Environmental Health Sciences. We support these data on its anti-cancerogenic effects with our results in HF-SAR cells. Aldehydes, such as 4-hydroxynonenal, seem to regulate the growth inhibition of Jurkat cells and increase the caspase-3 activity only at higher levels; therefore, we suggest that 5-HMF is able to react in a similar way [19]. Zhao et al. [18] presented 5-HMF as a potential cancer chemoprevention substance because of its reduction of reactive oxygen species and lipid peroxidation end products like malondialdehyde and its increase of antioxidatively acting enzymes like superoxide dismutase (SOD), catalase (CAT), and glutathione peroxidase (GPx).

The combined solution of aKG + 5-HMF resulted in a dose-dependent decrease in Jurkat cell proliferation. Additionally, this decrease also seemed to depend on time because the IC50% of aKG + 5-HMF after 72 h was about 100 µg/mL aKG + 41.7 µg/mL 5-HMF; it was 200 µg/mL aKG + 66.7 µg/mL after 48 h and 375 µg/mL aKG + 125 µg/mL 5-HMF after 24 h of incubation. It was reported that after 48 and 72 h, aKG resulted in a significant reduction in the proliferation in osteosarcoma cells—down to 80–90%—with the same aKG concentrations that we used [25]. While no similar results have been reported in the literature for Jurkat cells treated with 5-HMF, aKG could act against tumor cell proliferation by using glutaminolysis as a target. Cancer cells constantly encounter a variety of stress signals in vivo, such as reactive oxygen species (ROS) and nutrient limitations. Consequently, cancer cells must rewire their metabolic pathways under these environmental conditions. For example, mounting evidence suggests that cancer cells show elevated levels of ROS compared to normal cells. Glutaminolysis is directly involved in maintaining ROS homeostasis, which is crucial for cell growth and survival [26]. aKG itself showed an anti-oxidative potential by reducing peroxides, e.g., hydrogen peroxide and peroxynitrite, to tricarboxylic acid (TCA) cycle substances such as succinate and, as side products, nitrate and NO* [23].

aKG is involved in the requirement of carboxylation for mitochondrial defects in cancer cells [27] and, hence, in mitochondrial up- and downregulation. The anti-proliferative activity of 5-HMF was also shown in a cervical carcinoma cell line with methanol and water extracts from pyrrosia piloselloides, which revealed 5-HMF as a major component [28]. 

Here, AKG + 5-HMF showed great potential for decreasing the mitochondrial activity in Jurkat cells and, therefore, their proliferation. No mitochondrial activity effects were found in HF-SAR cells using aKG + 5-HMF, but a slight increase of cell proliferation was obtained using 500 µg/mL aKG + 166.7 µg/mL 5-HMF for 72 h combined with a reduction of carbonylated proteins in HF-SAR lysed cells. The experiments with on HF-SAR indicated that there were no cancerogenic effects of aKG and 5-HMF on normal healthy cells. 

The longer the incubation with Jurkat cells was the better the outcome of the reduction of mitochondrial activity was; it even reached down to 10% with the highest aKG+ 5-HMF concentration. The IC50% was assessed after 72 h of incubation in Jurkat cells with a lower aKG + 5-HMF concentration of 200 µg/mL aKG + 66.7 µg/mL 5-HMF, suggesting a synergistic effect of both substances in reducing peroxides [23] and activating antioxidative acting enzymes [9,18].

While there are no similar results for 5-HMF, aKG, and the combination thereof are available in the literature, the inactivation of caspase-9 with aKG was measured in osteosarcoma cells [25]. The usage of 10–50 mM aKG showed a significant dose-dependent increase in caspase activity of between 18 and 30% after 72 h of incubation in osteosarcoma cells.

After 72 h of incubation in Jurkat cells, the highest concentration of AKG + 5-HMF (500 µg/mL + 166.7 µg/mL) increased the caspase-3 activity by up to 51.6%, while 250 µg/mL aKG + 83.3 µg/mL 5-HMF increased it by up to 13.5%.

The calculated molarities of aKG used here were 3.4 and 1.7 mM, and these were 5 to 25-fold lower than the aKG concentrations used in the study with osteosarcoma. aKG might regulate caspase-3 activity in Jurkat cells at lower dosages than in osteosarcoma cells, but we suggest that 5-HMF had a greater impact on caspase-3 activation than aKG in the aKG + 5-HMF solution.

aKG is involved in autophagy, cell death, and cell apoptosis, which are regulated by p53 and oxoglutarate dehydrogenase [29], but 5-HMF is speculated to induce apoptosis. Here, we found a potential increase in activation of apoptosis of up to 63.2% with 500 µg/mL aKG + 166.7 µg/mL 5-HMF. In contrast, 5 mM aKG induced apoptosis in osteosarcoma cells after 72 h of treatment by only 7%, while 200 mM aKG increased apoptosis by 12.1%. 

Carbonylated proteins are a good and stabile marker for the detection of oxidative protein modification as a mechanism of redox signaling. Oxidative modification on proteins results from direct radical attack, like free radicals and RONS, or lipid peroxidation or glucose oxidized substances such as malondialdehyde, 4-hydroxy-nonenal. The oxidative modification takes place on specific amino acids, like cysteine or tyrosine, and can elicit changes in protein function and thereby mediating redox signaling, the so-called covalent redox signaling which differs from the classical receptor-ligand mediated signaling [30]. aKG + 5-HMF showed an effectively antioxidative reduction of carbonylated proteins in cell lysates of both used cell types. In Jurkat cells the effect was even higher because the content of carbonylated proteins was massively higher than to HF-SAR cells before incubation with aKG + 5-HMF. After 72 h incubation the carbonylated proteins were reduced nearly to half in Jurkat cells, but also in a lesser degree in HF-SAR.

## 5. Conclusions

Our data demonstrated that the combined solution of aKG + 5-HMF (1:3) showed an anti-proliferative impact on Jurkat cells in lowering mitochondrial-activity, increasing caspase-3-activity and apoptosis-inducing effects in Jurkat cells, which seems to be related to its antioxidative potential. However, no negative effect was obtained on HF-SAR in cell proliferation, whereas the mitochondrial activity was positively influenced. These results may thus provide a basis for further studies on cancer cells and on the individual compounds.

## Figures and Tables

**Table 1:** Cell proliferation (%) of the Jurkat cell line and of the HF-SAR after incubation for 0, 24, 48, and 72 h in the absence or presence of different combinations of aKG + 5-HMF solutions (*n* = 5).

Jurkat	0 h	24 h	48 h	72 h
Cell growth	%	%	%	%
0 µg/mL aKG + 0 µg/mL 5-HMF	100 ± 7.5	190.5 ± 13.6	286.0 ± 2.1	376.5 ± 5.0
125 µg/mL aKG + 41.7 µg/mL HMF	105 ± 3.5	150.5 ± 14.0	266.0 ± 6.0	331.0 ± 6.3
200 µg/mL aKG + 66.7 µg/mL 5-HMF	101 ± 2.5	168.1 ± 20.2	216.7 ± 5.8	304.5 ± 5.9
250 µg/mL aKG + 83.3 µg/mL 5-HMF	103 ± 9	165.5 ± 6.3	206.4 ± 7.8	290.5 ± 8.8
375 µg/mL aKG + 125 µg/mL 5-HMF	98.7 ± 5.5	179.0 ± 8.7	195.5 ± 4.6	296.5 ± 7.1
500 µg/mL aKG + 166.7 µg/mL 5-HMF	105 ± 3.5	165.5 ± 7.6	222.5 ± 3.4	256.0 ± 9.6
**HF-SAR**	**0 h**	**24 h**	**48 h**	**72 h**
Cell growth	%	%	%	%
0 µg/mL aKG + 0 µg/mL 5-HMF	101.3 ± 7.5	88.7 ± 9.9	92.3 ± 12.1	126.6 ± 14.5
125 µg/mL aKG + 41.7 µg/mL HMF	100.0 ± 3.1	85.0 ± 7.3	92.8 ± 9.2	121.7 ± 9.2
200 µg/mL aKG + 66.7 µg/mL 5-HMF	104.1 ± 1.5	89.3 ± 8.9	100.6 ± 7.9	121.4 ± 13.1
250 µg/mL aKG + 83.3 µg/mL 5-HMF	99.0 ± 6.1	82.7 ± 4.2	89.3 ± 4.1	137.6 ± 22.1
375 µg/mL aKG + 125 µg/mL 5-HMF	99.9 ± 3.5	93.8 ± 11.0	104.3 ± 11.1	132.6 ± 29.1
500 µg/mL aKG + 166.7 µg/mL 5-HMF	102.0 ± 3.1	86.2 ± 4.1	97.9 ± 8.2	140.5 * ± 23

**Table 2:** Mitochondrial activity (%) of the Jurkat cell line (A) and of the HF-SAR (B) after incubation for 24, 48, and 72 h in the absence or presence of different combinations of aKG + 5-HMF solutions (*n* = 5). * *p* < 0.01: significance between 24 h of incubation without aKG + 5-HMF and with the combined solutions of aKG + 5-HMF. ** *p* < 0.001: significance between 24 h of incubation without aKG + 5-HMF and with the combined solutions of aKG + 5-HMF. ^++^ *p* < 0.001: significance between 48 h of incubation without aKG + 5-HMF and with the combined solutions of aKG + 5-HMF. ^$$^ *p* < 0.001: significance between 48 h of incubation without aKG + 5-HMF and with the combined solutions of aKG + 5-HMF.

Jurkat	24 h	48 h	72 h
Mitochondrial Activity	%	%	%
0 µg/mL aKG + 0 µg/mL 5-HMF	100 ± 10.5	100 ± 4.4	100 ± 8.1
125 µg/mL aKG + 41.7 µg/mL HMF	90.5 ± 9.5	86.3 ± 14.6	83 ± 12.1
200 µg/mL aKG + 66.7 µg/mL 5-HMF	88.9 ± 7.9	63.7 ± 5.8 ^++^	49.5 ± 6.1 ^$$^
250 µg/mL aKG + 83.3 µg/mL 5-HMF	81.1 ± 15.8	52.9 ± 8.8 ^++^	44.4 ± 7.1 ^$$^
375 µg/mL aKG + 125 µg/mL 5-HMF	71.6 ± 9.5 *	35.1 ± 5.3 ^++^	18.2 ± 4.4 ^$$^
500 µg/mL aKG + 166.7 µg/mL 5-HMF	58.9 ± 11.1 **	28.7 ± 5.8 ^++^	9.9 ± 2.4 ^$$^
**HF-SAR**	**24 h**	**48 h**	**72 h**
Mitochondrial Activity	%	%	%
0 µg/mL aKG + 0 µg/mL 5-HMF	100 ± 11.4	100 ± 4.1	100 ± 8.4
125 µg/mL aKG + 41.7 µg/mL HMF	86.7 ± 5.3	90.7.3 ± 5.2	83.1 ± 6.2
200 µg/mL aKG + 66.7 µg/mL 5-HMF	90.7 ± 6.9	88.2 ± 8.3	91.1 ± 4.2
250 µg/mL aKG + 83.3 µg/mL 5-HMF	84.3 ± 5.1	85.3 ± 6.8	91.9 ± 5.1
375 µg/mL aKG + 125 µg/mL 5-HMF	90.0 ± 5.5	89.6 ± 6.1	95.8 ± 5.9
500 µg/mL aKG + 166.7 µg/mL 5-HMF	90.8 ± 4.6.1	83.7 ± 11.3	99.1 ± 9.2

**Table 3:** Carbonylated proteins of Jurkat and HF-SAR lysates after 0 and 72 h incubation in absence or presence of 500 µg/mL aKG + 125 µg/mL 5-HMF and 500 µg/mL aKG + 62.5 µg/mL 5-HMF (*n* = 5). * *p* < 0.05: significance between 0 h and 72 h incubated aKG + 5-HMF solutions. ** *p* < 0.01: significance between 0 h and 72 h incubated aKG + 5-HMF solutions. ^§§^ *p* < 0.01 = significance between Jurkat cell and HF-SAR lysate.

	Carbonylated Proteins (nmol/mg Protein)			
	Jurkat Lysate		HF-SAR Lysate	
	0 h	72 h	0 h	72 h
250 µg/mL aKG + 83.3 µg/mL 5-HMF	11.6 ^§§^ ± 0.67	7.44 ± 0.93 **	2.10 ± 0.72	1.91 ± 0.82
500 µg/mL aKG + 166.7 µg/mL 5-HMF	10.6 ^§§^ ± 0.37	5.55 ± 1.22 **	2.5 ± 0.6	1.73 ± 0.52 *

## Data Availability

The data presented in this study are available in article.

## References

[1] Greilberger J. (2013). Preclinical and clinical testing of a alpha-ketoglutarate/5-HMF/N-actyl-selenomethionine and N-acteyl-methionine for treating tumors. J. Cancer Sci. Ther..

[2] Herwig R., Horninger W., Pinggera G.M., Rehder P., Frauscher F., Konwalinka G.B. (2005). Metabolomics therapy with 2-oxo-glutaric acid solution (Karal solution) in patients with hormone and chemotherapy insensitive metastatic prostate cancer leads to an increase of PSA doubling time and decrease of blood supply in tumour lesions. Eur. Urol. Suppl..

[3] Greilberger J.F., Wintersteiger R., Astrid O., Greilberger M., Herwig R. (2018). Combination of 2-oxoglutarate/ascorbic acid/5-hydroxy-methyl-furfur-aldehyde/carnosine inhibits protein oxidation during radical exposure of cigarette smoke. Proteins.

[4] Kaelin W.G. (2011). Cancer and altered metabolism: Potential importance of hypoxia-inducible factor and 2-oxoglutarate-dependent dioxygenases. Cold Spring Harb. Symp. Quant. Biol..

[5] Losman J.A., Koivunen P., Kaelin W.G. (2020). 2020. 2-Oxoglutarate-dependent dioxygenases in cancer. Nat. Rev. Cancer.

[6] Yi W., Yu Y., Li Y., Yang J., Gao S., Xu L. (2021). The tumor-suppressive effects of alpha-ketoglutarate-dependent dioxygenase FTO via N6-methyladenosine RNA methylation on bladder cancer patients. Bioengineered.

[7] Ward P.S., Patel J., Wise D.R., Abdel-Wahab O., Bennett B.D., Coller H.A., Cross J.R., Fantin V.R., Hedvat C.V., Perl A.E. (2010). The common feature of leukemia-associated IDH1 and IDH2 mutations is a neomorphic enzyme activity converting alpha-ketoglutarate to 2-hydroxyglutarate. Cancer Cell.

[8] Shapla U.M., Solayman M., Alam N., Khalil M.I., Gan S.H. (2018). 5-Hydroxymethylfurfural (HMF) levels in honey and other food products: Effects on bees and human health. Chem. Cent. J..

[9] Zhao L., Su J., Li L., Chen J., Hu S., Zhang X. (2014). Mechanistic elucidation of apoptosis and cell cycle arrest induced by 5-hydroxymethylfurfural, the important role of ROS-mediated signaling pathways. Food Res..

[10] Bito T., Koseki K., Asano R., Ueda N. (2020). 5-hydroxymethyl-2-furaldehyde purified from Japanese pear (*Pyrus pyrifolia* Nakai cv. Nijisseiki) juice concentrate inhibits melanogenesis in B16 mouse melanoma. Bioscience.

[11] Kössler F., Mair L., Burtscher M. (2019). 5-Hydroxymethylfurfural and α-ketoglutaric acid supplementation increases oxygen saturation during prolonged exercise in normobaric hypoxia. Int. J..

[12] Kassa T., Wood F., Strader M.B., Alayash A.I. (2019). Antisickling Drugs Targeting βCys93 Reduce Iron Oxidation and Oxidative Changes in Sickle Cell Hemoglobin. Front. Physiol..

[13] Levine R.L., Williams J.A., Stadtman E.R., Shacter E. (1994). Carbonyl assays for determination of oxidatively modified proteins. Methods Enzymol..

[14] Curnock A.P., Thomson T.A., Westwood R., Kuo E.A., Williamson R.A., Yea C.M., Ruuthb E. (2001). Inhibition of stimulated Jurkat cell adenosine 3’,5’-cyclic monophosphate synthesis by the immunomodulatory compound HR325. Biochem. Pharmacol..

[15] Wolf C., Lederer K., Pfragner R., Schauenstein K., Ingolic E., Siegl V. (2007). Biocompatibility of ultra-high molecular weight polyethylene (UHMW-PE) stabilized with alpha-tocopherol used for joint endoprostheses assessed in vitro. J. Mater. Sci. Mater. Med..

[16] Hong S.H., Ismail I.A., Kang S.M., Han D.C., Kwon B.M. (2016). Cinnamaldehydes in Cancer Chemotherapy. Phytother. Res..

[17] Stuart S.D., Schauble A., Gupta S., Kennedy A.D., Keppler B.R., Bingham P.M., Zachar Z. (2014). A strategically designed small molecule attacks alpha-ketoglutarate dehydrogenase in tumor cells through a redox process. Cancer Metab..

[18] Zhao L., Chen J., Su J., Li L., Hu S., Li B., Zhang X., Xu Z., Chen T. (2013). In vitro antioxidant and antiproliferative activities of 5-hydroxymethylfurfural. J. Agric. Food Chem..

[19] Larini A., Bianchi L., Bocci V. (2004). Effect of 4-hydroxynonenal on antioxidant capacity and apoptosis induction in Jurkat T cells. Free Radic. Res..

[20] Cobbs C.S., Whisenhunt T.R., Wesemann D.R., Harkins L.E., van Meir E.G., Samanta M. (2003). Inactivation of wild-type p53 protein function by reactive oxygen and nitrogen species in malignant glioma cells. Cancer Res.

[21] Li X., de Sarno P., Song L., Beckman J.S., Jope R.S. (1998). Peroxynitrite modulates tyrosine phosphorylation and phosphoinositide signalling in human neuroblastoma SH-SY5Y cells: Attenuated effects in human 1321N1 astrocytoma cells. Biochem. J..

[22] Long L.H., Halliwell B. (2011). Artefacts in cell culture: α-Ketoglutarate can scavenge hydrogen peroxide generated by ascorbate and epigallocatechin gallate in cell culture media. Biochem. Biophys. Res. Commun..

[23] Greilberger J., Greilberger M., Wintersteiger R., Zangger K., Herwig R. (2021). Alpha-Ketoglutarate: A Potential Inner Mitochondrial and Cytosolic Protector against Peroxynitrite and Peroxynitrite-Induced Nitration. Antioxidants.

[24] Long L.H., Halliwell B. (2012). The effects of oxaloacetate on hydrogen peroxide generation from ascorbate and epigallocatechin gallate in cell culture media: Potential for altering cell metabolism. Biochem. Biophys. Res. Commun..

[25] Kaławaj K., Sławińska-Brych A., Mizerska-Kowalska M., Żurek A., Bojarska-Junak A., Kandefer-Szerszeń M., Zdzisińska B. (2020). Alpha Ketoglutarate Exerts In Vitro Anti-Osteosarcoma Effects through Inhibition of Cell Proliferation, Induction of Apoptosis via the JNK and Caspase 9-Dependent Mechanism, and Suppression of TGF-β and VEGF Production and Metastatic Potential of Cells. Int. J. Mol. Sci..

[26] Jin L., Alesi G.N., Kang S. (2016). Glutaminolysis as a target for cancer therapy. Oncogene.

[27] Mullen A.R., Hu Z., Shi X., Jiang L., Boroughs L.K., Kovacs Z., Boriack R., Rakheja D., Sullivan L.B., Linehan W.M. (2014). Oxidation of alpha-ketoglutarate is required for reductive carboxylation in cancer cells with mitochondrial defects. Cell Rep..

[28] Sul’ain M.D., Zakaria M.F.J.F. (2019). Anti-Proliferative Effects of Methanol and Water Extracts of Pyrrosia piloselloides on the Hela Human Cervical Carcinoma Cell Line. Asian Pac. J. Cancer Prev..

[29] Duan L., Perez R.E., Maki C.G. (2019). Alpha ketoglutarate levels, regulated by p53 and OGDH, determine autophagy and cell fate/apoptosis in response to Nutlin-3a. Cancer Biol. Ther..

[30] Wall S.B., Oh J.Y., Diers A.R., Landar A. (2012). Oxidative modification of proteins: An emerging mechanism of cell signaling. Front. Physiol..

